# Population structure in chicory (*Cichorium intybus*): A successful U.S. weed since the American revolutionary war

**DOI:** 10.1002/ece3.2994

**Published:** 2017-05-02

**Authors:** Tomáš Závada, Rondy J. Malik, Rick V. Kesseli

**Affiliations:** ^1^ Biology DepartmentUniversity of Massachusetts BostonBostonMAUSA; ^2^ Present address: Sterling CollegeCraftsbury CommonVTUSAand; ^3^ Present address: Department of Ecosystem Science and ManagementPennsylvania State UniversityUniversity ParkPAUSA

**Keywords:** Asteraceae, chicory, *Cichorium intybus*, genetic diversity, population genetics, weed

## Abstract

Plant invasions are recognized as major drivers of ecosystem change, yet the precise cause of these invasions remains unknown for many species. Frequency and modes of introductions during the first, transport and colonization, stages of the invasion process as well as phenotypic changes due to plasticity or changing genetic diversity and adaptation during later establishment and expansion stages can all influence the “success” of invasion. Here, we examine some of these factors in, and the origin of, a very successful weed, *Cichorium intybus* (chicory) which was introduced to North America in the 18th century and which now can be found in all 48 continental U.S. states and much of Canada. We genotyped a Eurasian collection of 11 chicory cultivars, nine native populations and a North American collection of 20 introduced wild populations which span the species range (592 individuals in total). To detect the geographic sources of North American chicory populations and to assess the genetic diversity among cultivars, native, and introduced populations, we used both a sequenced cpDNA region and 12 nuclear simple sequence repeat (SSR), microsatellite loci. Four cpDNA haplotypes were identified and revealed clear geographic subdivisions in the chicory native range and an interspecific hybrid origin of Radicchio group. Nuclear data suggested that domesticated lines deliberately introduced to North America were major contributors to extant weedy populations, although unintended sources such as seed contaminants likely also played important roles. The high private allelic richness and novel genetic groups were detected in some introduced populations, suggesting the potential for local adaptation in natural sites such as deserts and nature reserves. Our findings suggest that the current populations of weedy U.S. chicory have evolved primarily from several sources of domesticated and weedy ancestors and subsequent admixture among escaped lineages.

## 1 Introduction

Earnest interest in the potential ecological and evolutionary importance of non‐native species began more than 50 years ago by biologists with many different perspectives (Elton, 1958; Harper, 1960; Baker & Stebbins, 1965). Much has been studied, discussed, and written since that time, but the data seem clear; these species can evolve quickly (Colautti & Barrett, 2013) and, for those non‐native species that have successfully expanded their ranges, their impacts on local ecosystems are largely negative (Mack et al., 2000; Vilà et al., 2011). The pathways from introduced resident to weedy invader are, however, varied, and few taxa complete this process and become problematic and noxious (Theoharides & Dukes, 2007; Williamson & Fitter, 1996). Certain life‐history traits of a species make this transition more likely and features such as short generation time, fast growth, developmental plasticity, resistance to environmental stress, predation and disease, high and consistent reproductive output, small seeds, and variable seed dormancy have all been identified as common in invasive species (Rejmánek & Richardson, 1996). Despite these suits of traits common to many invaders, the possible combinations of “weedy” traits are many, the exceptions are common and predictions of which species might become invasive based on the presence of some set of traits are difficult. Clearly other factors also influence the outcome of these events.

Factors such as the number and source of introductions into new environments have also proven to be important and often are a consequence of human socioeconomic activities. Most noxious invaders have shown a lag time between first introductions, establishment, and the range expansion. With historical data, Aikio, Duncan, and Hulme (2010) quantified this effect showing that biased sampling was not responsible for the lag. Forman (2003) showed that species with five or more vectors of introduction were significantly more likely to fall into the nonbenign invasive category than species with few modes of introduction. In addition, many other studies indicate that successful invasions are associated with multiple introductions and subsequent intra‐ or interspecific hybridization (Ellstrand & Schierenbeck, 2006; Grimsby, Tsirelson, Gammon, & Kesseli, 2007; Simberloff, 2009). Genetic admixture may benefit invaders in two ways; first, by increasing allelic diversity which provides a larger pool of raw material; and second, by generating unique allele and gene combinations which create novel phenotypes (Lavergne & Molofsky, 2007). The implication is that species need time to accumulate genetic diversity, to adapt, and to evolve in the new environments and that number, mode, source, and time span of introductions may be important factors affecting this lag time and contributing to the evolutionary potential and success of a species (Bossdorf et al., 2005; Callaway & Maron, 2006; Parker et al., 2013).

Domesticated plants have often been introduced many times and have been selected for disturbed, anthropogenic, albeit controlled, habitats (Mack et al., 2000; Reichard & White, 2001). This clearly increases the opportunities for escape and while not always increasing the probability of success at later stages of an invasive pathway (Williamson & Fitter, 1996), domesticated taxa are likely to become more problematic in the future considering the expanding horticultural industry as it adapts to changing preferences and needs of human populations (Bradley et al., 2012). *Cichorium intybus* (chicory) is an established, cosmopolitan weed. It is diploid (2n = 18), perennial, self‐incompatible, and possesses extensive phenotypic diversity. Chicory is grown for its roots which are used as a coffee surrogate, a source of polysaccharide inulin, and as a leafy vegetable (Kiers, Mes, Van der Meijden, & Bachmann, 1999). Chicory is native to Eurasia and the majority of the world production and breeding is in European countries. Most of the U.S. commercially produced chicory comes from California, New York, and Ohio (www.nass.usda.gov/Data_and_Statistics/). Chicory also became a weedy/invasive species in North America and Australia and is labeled a noxious weed in the state of Colorado. Weedy chicory can be found across North America in 48 continental states of the United States and most provinces of Canada (USDA Plants Database). Chicory was also collected in 1956 on O'ahu Island (www.hear.org/vouchers/pier/bish0000011844.htm), but is not currently reported in Hawaii. Chicory exhibits a great range of phenotypes for leaf shape, color, leaf surface, hairiness, as well as plant size and, based on greenhouse experiments with variable soil types, temperatures and climatic conditions much of the phenotypic diversity can be attributed to environmental plasticity (Gemeinholzer & Bachmann, 2005). The plasticity of this species has been discussed for more than a century and was noted by early American farmers in field observations “…the foliage [of chicory cultivars] is by no means a constant character of variety” (Kains, 1898).

Five chicory cultivar groups are distinguished (Van Stallen, Noten, Neefs, & de Proft, 2001; http://ecpgr.cgn.wur.nl/lvintro/): *var. sativum* (1) Root chicory, and the remaining groups used for leaves: *var. foliosum* (2) Witloof (or Belgian endive), (3) Pain de Sucre, (4) Radicchio, and (5) Catalogna. All the red types of radicchio are believed to come from red‐leafed *var. foliosum*, while plants with spotted or variegated leaves likely originated from spontaneous or controlled crosses between red‐leafed chicory *var. foliosum* and broadleaved endive *Cichorium endivia* (Barcaccia et al., 2003). Chicory is primarily cultivated in the Mediterranean region (Zeven, 1982). The oldest archaeological evidence of the use of *C. intybus* dates from the Bronze Age and it has been found at the Alpenquai site in Zurich, Switzerland (Smartt & Simmonds, 1995).

AFLP and RAPD markers for chicory were developed during the last two decades (Bellamy, Vedel, & Bannerot, 1996; Kiers, Mes, van der Meijden, & Bachmann, 2000; Koch & Jung, 1997; Van Cutsem et al., 2003; Van Stallen et al., 2001). Some of these markers have been used to construct a genetic map of chicory that was based on an intraspecific F2 population derived from a cross between two inbred lines of Witloof chicory varieties (Van Stallen, Vandenbussche, Verdoodt, & De Proft, 2003). Cadalen et al. (2010) constructed a consensus genetic map for chicory after the integration of molecular marker data of two industrial chicory progenies and one Witloof chicory progeny. These genetic markers have been useful for elucidating the origins and evolutionary history of the various domesticated lines. The genetic variation of available Witloof cultivars was shown to be low using RFLP data (Bellamy, Mathieu, Vedel, & Bannerot, 1995). In contrast, radicchio cultivars are highly heterozygous and genetically heterogeneous with some lines originating from a cross between *C. intybus* and *C. endivia* (Van Stallen et al., 2001). Unlike the situation for many domesticated species, particularly inbred taxa, most of genetic variation in the radicchio cultivars is partitioned within not between accessions (Barcaccia et al., 2003). Kiaer et al. (2009) measured spontaneous gene flow among wild European and cultivated chicory. The study indicated high levels of gene flow among populations in Europe with many incidents of recent gene flow between cultivars and wild populations.

The invasion history of chicory in North American is mostly unknown although there are some fascinating anecdotal accounts. One of the first records of planting chicory in the United States can be found in Thomas Jefferson's correspondence and dates back to 1774. Jefferson's garden book in Monticello showed that he sowed “Radiccio di Pistoia” on 15 March 1774. Arthur Young carried chicory seeds from France to England and sent some seeds to General Washington, who gave some to Jefferson. In 1785, Governor James Bowdoin of Massachusetts had chicory planted in his fields to feed sheep; the seeds came from Holland. By 1818, it was abundant around Philadelphia, according to one of the pioneers of American medicinal botany, Dr. William Barton. The future success of this species in colonizing the United States was indicated in Jefferson's 1811 letter “…[*Sichorium Intibus*] has been growing here in abundance and perfection now 20 years without any cultivation after the first transplanting” (Looney, 2004). Chicory plants would start to spread all over the continental United States to the point, that by 1900s, farmers would call for a chicory control and eradication. Seeds were distributed as an impurity in both foreign and domestic grass and clover seed (Hansen, 1920).

Population genetic structure can reveal some aspects of the invasion history of a species, most notably sources and modes of introductions and hybridization events (Fitzpatrick, Fordyce, Niemiller, & Reynolds, 2012), which should provide a more complete understanding of invasive weeds and enable better management of invasions. Considering the references to chicory, both as a grass and clover seed contaminant, and as a crop, it is very likely that the invasion history of this species in North American is complicated. Currently, the levels of diversity, likely number and sources of introduction, occurrence of hybridization and the importance of selection are all unknown factors which may have affected the invasion process of chicory in North America. In this study, we genotyped cultivars, as well as wild Eurasian and North American chicory populations in order to assess the genetic diversity of this species, and to examine evolutionary changes since chicory was introduced to the United States in the late 1700s.

## 2 Methods

### 2.1 Plant material

**Figure 1:**
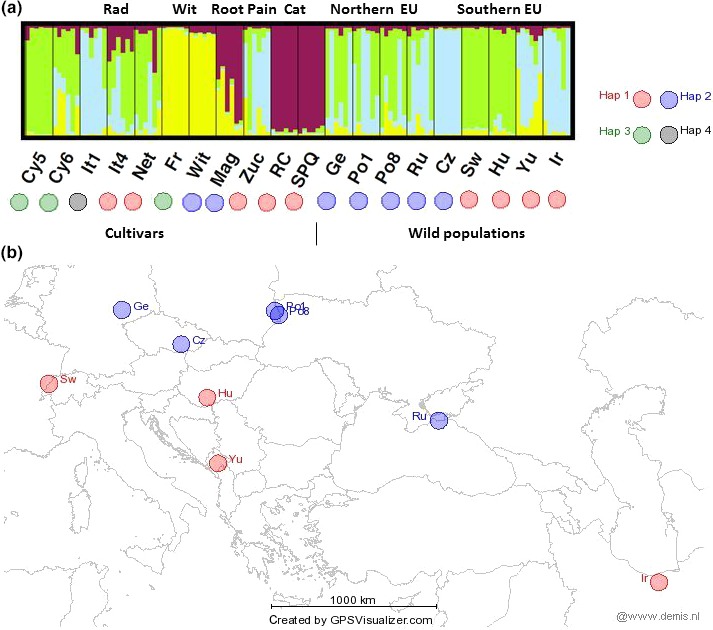
Inference of population structure in the Eurasian collection of 11 cultivars (left side populations Cyp5—SPQ) and nine wild accessions (right side, populations Ge—Ir) of chicory. (a) STRUCTURE analysis of the 20 accessions each separated by a black bar and based on 12 SSR markers with *K* = 4 and the cpDNA haplotype (Hap 1–4) defined by a color‐coded circle below each accession. (b) Geographical distribution of cpDNA haplotypes in the nine wild Eurasian populations. Hap 1—red, Hap 2—blue, Hap 3—green, Hap 4—black. Accession abbreviations can be found in Table 1

**Figure 2:**
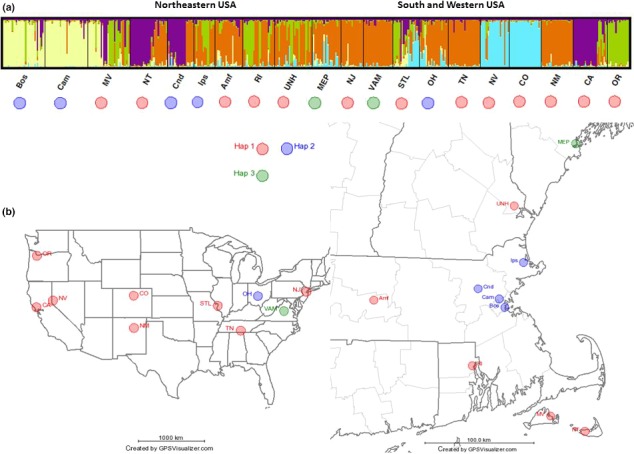
Inference of population structure in the North American collection of 20 wild chicory populations. (a) STRUCTURE analysis of the populations each separated by a black bar and based on 12 SSR markers with *K* = 5. The cpDNA haplotypes with color coding as defined in Figure 1 are shown under each accession. (b) Geographical distribution of cpDNA haplotypes. Accession abbreviations can be found in Table 2

Our “Eurasian collection” consists of 11 domesticated lines and nine wild accessions obtained from a variety of sources and grown from seed in our greenhouse at University of Massachusetts, Boston (Table 1 and Figure 1). Our “North American collection” is derived from leaf samples collected from 20 wild populations across North America during the summers of 2011, 2012, and spring of 2014, with most from the eastern regions and others scattered as far west as California (see Table 2 and Figure 2 for locations and source of the collections). For the assays, we scored between 6 and 32 random plants per population, for a total of 592 individuals.

**Table 1:** Chicory cultivars (1–11) and wild (12–20) Eurasian chicory populations

Population	*N*	Group	USDA/Accession	Cultivar type/Origin, GIS Coordinates	*H*_o_	*H*_e_	*F*	cpDNA haplotype
1. Cy5	6	C	PI 432335	Salata—primitive cultivar, Cyprus	0.500	0.603	0.171	3
2. Cy6	6	C	PI 432336	Salata—primitive cultivar, Cyprus	0.556	0.663	0.162	3
3. It1	6	C	PI 651961	Radicchio—”Variegata Di Chioggia”	0.653	0.756	0.137	4
4. It4	6	C	PI 652048	Radicchio—”Variegata Di Chioggia”	0.639	0.708	0.098	1
5. Net	6	C	PI 651886	Radicchio—”Augusto”	0.561	0.702	0.202	1
6. Fr	6	C	PI 652017	Witloof—”Turbo”	0.383	0.439	0.128	3
7. Wit	6	C	PI261776	Witloof—”Chicoree de Bruxelles”	0.389	0.508	0.234	2
8. Mag	6	C	Stokes	Root—”Magdeburgh”	0.500	0.533	0.062	2
9. Zuc	6	C	PI 651954	Pain de Sucre—”Zuckerhut”	0.528	0.648	0.185	1
10. RC	6	C	Stokes	Catalogna—”Radichetta”	0.500	0.579	0.136	1
11. SPQ	6	C	Stokes	Catalogna—”Cicoria San Pasquale”	0.667	0.647	−0.030	1
12. Ge	6	W	PI 652006	Zangenberg, Germany 51.066, 12.150	0.611	0.708	0.137	2
13. Po1	6	W	PI 652034	Chelm, Poland 51.016, 23.666	0.697	0.740	0.058	2
14. Po8	6	W	PI 652009	Zamosc, Poland 50.783, 23.95	0.530	0.612	0.133	2
15. Ru	6	W	PI 652028	Krasnodar, Russia 45.032, 35.976	0.625	0.694	0.100	2
16. Cz	6	W	Wild	Brno, Czech Republic 49.195,16.606	0.625	0.710	0.119	2
17. Sw	6	W	PI 652019	Switzerland 47.039, 6.65	0.567	0.556	−0.019	1
18. Hu	6	W	PI 531292	Borzsony, Hungary 46.288,18.56	0.591	0.652	0.093	1
19. Yu	6	W	PI 652030	Montenegro, 42.708,19.374	0.542	0.616	0.121	1
20. Ir	6	W	PI 652026	Mazandaran, Iran 36.226, 52.531	0.556	0.635	0.125	1

*N*, sample size; *H*_o_, average observed heterozygosity; *H*_e_, average expected heterozygosity; *F*, inbreeding coefficient; W, collected in wild; C, cultivar.

**Table 2:** North American chicory populations

Population	*N*	Location	GIS Coordinates	*H*_o_	*H*_e_	*F*	cpDNA haplotype
1. Bos	32	Boston, MA	42.306, −71.049	0.500	0.642	0.221	2
2. Cam	32	Cambridge, MA	42.377, −71.111	0.424	0.581	0.269	2
3. MV	32	Martha's Vineyard, MA	41.408, −70.536	0.418	0.644	0.352	1
4. NT	28	Nantucket, MA	41.280, −70.149	0.484	0.636	0.240	1
5. Cnd	20	Concord, MA	42.460, −71.348	0.447	0.614	0.272	2
6. Ips	16	Ipswich, MA	42.678, −70.840	0.498	0.600	0.170	2
7. Amf	21	Amherst, MA	42.366, −72.516	0.615	0.669	0.081	1
8. RI	24	Providence, RI	41.823, −71.412	0.510	0.664	0.231	1
9. UNH	28	Durham UNH, NH	43.146, −70.944	0.565	0.684	0.174	1
10. MEP	22	Portland, ME	43.661, −70.255	0.531	0.617	0.139	3
11. NJ	17	Woodbridge, NJ	40.557, −74.284	0.573	0.576	0.004	1
12. VAM	22	Monticello, VA	37.915, −78.326	0.631	0.630	−0.002	3
13. STL	20	St. Louis, MO	38.627, −90.199	0.595	0.719	0.172	1
14. OH	22	Columbus, OH	39.961, −82.998	0.487	0.598	0.186	2
15. TN	24	Decherd, TN	35.236, −86.071	0.462	0.570	0.108	1
16. NV	22	Pleasant Valley, NV	39.360, −119.763	0.618	0.600	−0.029	1
17. CO	24	Boulder, CO	40.014, −105.270	0.428	0.472	0.094	1
18. NM	24	Park Springs Ranch, NM	35.593, −105.223	0.453	0.516	0.122	1
19. CA	26	Santa Rosa, CA	38.440, −122.714	0.482	0.592	0.186	1
20. OR	16	Portland, OR	45.482, −122.630	0.472	0.580	0.187	1

*N*, sample size; *H*_o_, average observed heterozygosity; *H*_e_, average expected heterozygosity; F, inbreeding coefficient.

### 2.2 Markers and genotyping

In this study, we used twelve microsatellite nuclear markers (Table 3) and the sequencing of one uniparentally inherited chloroplast *trnL*‐*trnF* region (Taberlet, Gielly, Pautou, & Bouvet, 1991) to detect the geographic sources of North American chicory populations. We screened 41,704 ESTs derived from *Cichorium intybus* and 30,170 ESTs derived from *Cichorium endivia in* the Composite Genome Project database http://cgpdb.ucdavis.edu/asteraceae_assembly in order to identify all 3‐bp simple sequence repeats (SSRs) with 10 or more repeats, and 4‐bp SSRs with 5 or more repeats. Primers flanking the SSRs were designed in Primer3 program (Rozen & Skaletsky, 2000). We attached a 17‐bp M13 tag onto the 5′end of the forward primer and then tested 30 primer pairs for polymorphism. All markers were scored for the presence of linkage disequilibrium (LD) between microsatellite loci using GENEPOP (http://genepop.curtin.edu.au), but no evidence for LD was detected. The final set of twelve most polymorphic markers for *C. intybu*s was employed for genotyping all the individuals.

**Table 3:** Microsatellite markers for the genus *Cichorium*

Locus	NCBI sequence	Alleles	Repeat motif in library	Primer sequence (5′–3′)	Size range (bp)
5291	CCIL5291.b1_F04.ab1	24	(AAG)_16_	F: M13‐GCATCCACTCAAGCTCATTG	156–273
				R: TGGATTTCTAGGCCACACCT	
3984	CCIM3984.b1_P11.ab1	8	(AAG)_11_	F: M13‐GCAGCAACAACCCTTTCTTT	204–225
				R: GGTGGCGATTGAATTGAAGA	
5055	CCIS5055.b1_M15.ab1	15	(CAA)_10_	F: M13‐TGTGAGACGTGGGATTCTGA	213–291
				R: GCTTTGGCTCCCTATGTCAC	
12770	CCIM12770.b1_D01.ab1	15	(CTT)_18_	F: M13‐ CATAAAGGCCCTCCATTCCAC	168–237
				R: GTAAAGCCAAGCGAGACAGG	
6865	CCIL6865.b1_B14.ab1	10	(GAT)_10_	F: M13‐AAATGGTTCTGCATCAAAGGA	231–258
				R: CGATGGGGCTTGTTTCTTTA	
1385	CCIL1385.b1_A12.ab1	26	(GAT)_11_	F: M13‐TTGCCTCTTGCTCCAATACC	144–225
				R: GGGTCCCTTTGTGTCATCAT	
11019	CCEL11019.b1_E20.ab1	11	(ATTA)_5_	F: M13‐CAATCGGTTAATCAATCAAATCAA	219–291
				R: GGTATCGTAAGCCAGCCAAA	
13676	CCEL13676.b1_G12.ab1	14	(CAC)_10_	F: M13‐TCAACGTGCTTCAAGACGAC	225–270
				R: GTGGTGGTGGTTCGACTTTT	
2050	CCIS2050.b1_D09.ab1	9	(CTT)_10_	F: M13‐GCAACGGATGAAGGGTTACA	186–210
				R: GGAAATTAACCCCGGAAAAA	
3899	CCEL3899.b1_E15.ab1	9	(AATC)_5_	F: M13‐CCTCGACAGAAAACCCTCTTC	207–228
				R: AGGTGCGGAAGCGTAAGTT	
7179	CCIS7179.b1_E20.ab1	11	(CTT)_10_	F: M13‐GGCAGGACGTCTTTTTGGTA	186–225
				R: CCGAAGAATTTGAGGTTTG	
**8271**	CCEM8271.b1_M04.ab1	10	(ATG)_11_	F: M13‐AACAATGGTGGGCAGAAAAC	156–201
				R: CAGGGGTAAATCGGGAAAAT	

Seeds for chicory cultivars and Eurasian populations were grown in the greenhouse, and leaf tissue was harvested for DNA extractions. North American chicory DNA was extracted from dried leaves. Collectors dried partial or full leaves from flowering chicory populations either by placing them into 15 ml tubes containing DriRite (W.A. Hammond Drierite Co. Ltd., Xenia, OH, USA) or by pressing the leaves in newspaper. Samples were mailed to authors together with population location information. FastDNA extraction kit (MP Biomedicals, Solon, Ohio, USA) was used for DNA extractions according to manufacturer's protocol. Chloroplast primers were used as described by Taberlet et al. (1991). PCRs with microsatellite markers were performed in 25 μl volume with 5 μl of diluted DNA (20–100 ng), 0.25 μl of the forward primer, 0.75 μl of the fluorescently labeled M13 primer, and 1 μl of the reverse primer (each primer at10 pmol/μl), 5.0 μl of 5× reaction buffer, 2.5 μl of 2.5 mmol/L combined dNTPs, 2.5 μl of 25 mmol/L MgCl_2_, and 0.2 μl of *Taq* polymerase. The final reaction volume was brought to 25 μl with sterile water. We used a touchdown protocol with following cycles: 5‐min denaturation at 95°C, ten cycles of 30 s at 94°C, 30 s at 60°C, and 45 s at 72 °C, annealing temperature decreasing to 50°C by 1°C per cycle, followed by 30 cycles of 30 s at 94°C, 30 s at 50°C for 30 s, 45 s at 72°C for 30 s, followed by a final extension at 72°C for 5 min.

### 2.3 Data analysis

Chloroplast DNA (cpDNA) fragments were sequenced and PCR products targeting microsatellite regions were assayed on the 3100‐*Avant* sequencer (Applied Biosystems, Foster City, California, USA). cpDNA sequence editing and alignment was performed using the program Sequencher 4.9 (http://genecodes.com/). We used Peak Scanner software for microsatellite fragment length scoring (Applied Biosystems). Peaks were assigned numbers by Peak Scanner based on the 400HD Rox size ladder which approximated the length of amplicons. Each individual peak size was confirmed visually. The observed (*H*_o_) and the expected (*H*_e_) heterozygosity, inbreeding coefficient (*F*), and the analysis of molecular variance (AMOVA) were calculated using Arlequin v. 3.5.1.3 (Excoffier, Laval, & Schneider, 2005). Significance of Φ_ST_ values was determined via the maximum number of permutations in Arlequin 3.5. To characterize the genetic diversity at the population level and to control for sample size variation, allelic richness and private allelic richness were calculated using a rarefaction method in HP‐Rare (Kalinowski, 2005). Chloroplast DNA haplotype maps were constructed using GPS visualizer (http://www.gpsvisualizer.com/).

The ancestry and the genetic composition of chicory individuals were evaluated with a Bayesian clustering method in program Structure v. 2.3.4 (Falush, Stephens, & Pritchard, 2003; Pritchard, Stephens, & Donnelly, 2000). We assumed that all loci were independent and found no evidence of linkage disequilibrium using Arlequin v. 3.5. All individuals were allowed to be products of admixture, and we used prior information about the population origin. The length of burn‐in period was set to 200,000 iterations, and the number of Markov chain Monte Carlo (MCMC) steps after burn‐in was 1,000,000. We conducted five independent runs with a partial data set (120 individuals—11 chicory cultivars and 9 wild chicory Eurasian chicory populations, with *K* set from 1 to 7), and with a complete data set (592 individuals) with *K* set from 1 to 10, with 10 iterations for each *K* in each independent run. Structure results were run through STRUCTURE HARVESTER v. 0.6.93 (Earl & vonHoldt, 2012) in order to calculate Δ*K* for each value of *K* according to Evanno, Regnaut, and Goudet (2005). The STRUCTURE HARVESTER output data were permuted with CLUMPP v. 1.1.2 (Jakobsson & Rosenberg, 2007). The final visualization of genetic data was plotted with DISTRUCT v. 1.1 (Rosenberg, 2004).

## 3 Results

### 3.1 Chloroplast markers

Two random samples from each population were sequenced at *trnL*‐*trnF* locus. We detected four cpDNA haplotypes (Table 4) that were 702–716 bp long. Two haplotypes detected in Eurasian wild populations (EU) revealed a strong geographic differentiation in the native range of chicory (Figure 1). All northern populations (Ge, Po1, Po8, Ru, Cz) shared haplotype 2 (Hap 2) and southern populations (Sw, Hu, Yu, Ir) shared another haplotype (Hap 1). We found four haplotypes in chicory cultivar (CC) group, none of them diagnostic for a certain cultivar type. Hap 1 (southern wild populations) was detected in five cultivar accessions—in Radicchio group (It4, Net), in Pain de Sucre group (Zuc) and in both Catalogna accessions (RC, SPQ). Hap 2 (northern wild populations) was found in two cultivar types—one in Root (Mag) and one in Witloof group (Wit). Haplotype 3 (Hap 3) was detected in two primitive cultivar accessions (Cy5 and Cy6) and in one in Witloof group (Fr). Haplotype 4 (Hap 4) was found only in one Radicchio accession (It4) and the sequence contained an 11‐bp indel of AAAGAATTAGG. After being BLASTed against NCBI database, Hap 4 matched the common *Cichorium endivia* (endive) haplotype. None of the North American chicory populations (NA) possessed more than one haplotype in our analysis. Hap 1 was the most common and found in 13 of 20 populations. Hap 2 was detected in five populations (Bos, Cam, Cnd, Ips, and OH) and Hap 3 in two populations (MEP, Vam) in North America (Figure 2).

**Table 4:** Haplotype assignment based on cpDNA sequences

cpDNA haplotype	GenBank accession	SNP/INDEL positions					Populations
		67–69	216	318–328	582	678	
Hap1	KF879574	AGC	G	–	G	T	It4, Net, Zuc, RC, SPQ, Sw, Hu, Yu, Ir, MV, NT, Amf, RI, UNH, NJ, STL, TN, NV, CO, NM, CA, OR
Hap2	KF879575	–	A	–	G	T	Wit, Mag, Ge, Po1, Po8, Ru, Cz, Bos, Cam, Cnd, Ips, OH
Hap3	KF879576	AGC	G	–	C	T	Cy5, Cy6, Fr, MEP, Vam
Hap4	KF879577	AGC	G	AAAGAATTAGG	G	G	It1

### 3.2 Nuclear markers

Twelve assayed microsatellite loci were polymorphic, and markers amplified in all 592 individuals. The number of alleles per locus ranged from 8 to 26 (Table 3). The expected heterozygosity or gene diversity (*H*_e_) of all populations was generally high ranging from 0.44 to 0.76 (Tables 1 and 2), but the means for the three groups CC (*H*_e_ = 0.617), EU (*H*_e_ = 0.658), and NA (*H*_e_ = 0.610) were similar, although slightly higher in the EU group (Table 5). The domesticated CC lines when compared to the wild EU, for which samples sizes were equivalent, possessed substantial levels of genetic diversity but were generally less polymorphic, had fewer alleles per locus, and higher inbreeding coefficients. The most extreme case was Witloof cultivar “Chicoree de Bruxelles” which was monomorphic for half its loci, carried the fewest alleles, and had the highest inbreeding coefficient (*F* = 0.23) among all CC and EU accessions (Table 1). Several North American populations (Table 2) also had relatively high inbreeding coefficients. The average allelic richness, standardized for sample size differences, was lower in the domesticated CC accessions (3.31) than and in native, EU (3.84) and invasive, NA (3.79) populations (Table 5). The highest private allelic richness was detected in introduced populations (Nevada and New Mexico populations). All North American populations, except for one (Ips), had private alleles (Appendix S1), but only four of these alleles exceeded a frequency of 0.10 in the given population and the highest frequency was 0.27 for one allele in TN.

**Table 5:** Mean genetic diversity statistics comparing wild Eurasian chicory, cultivars, and introduced North American populations

Population	*N*	%P	AL	PAL	*H*_o_	*H*_e_
Native Eurasian	54	96.26	3.84	0.011	0.594	0.658
Cultivars	66	89.39	3.31	0.043	0.532	0.617
Introduced North American	472	98.74	3.79	0.077	0.513	0.610

*N*, number of plants sampled; %P, percent polymorphic loci; AL, allelic richness (based on the rarefaction method) averaged across all loci; PAL, private allelic richness (based on the rarefaction method) averaged across all loci; *H*_o_, observed heterozygosity averaged across all loci; *H*_e_, expected heterozygosity averaged across all loci.

Pairwise *F*_ST_ values between cultivars, Eurasian, and North American chicory groups were low but significant (*p* < .05; nonparametric permutation test; Excoffier et al. 1992), suggesting high intergroup gene flow; not surprising for a highly outcrossing species. Interestingly, the domesticated cultivars (CC) had significantly lower *F*_ST_ values with both the wild native EU (*F*_ST_ = 0.0254) and the wild invasive NA (*F*_ST_ = 0.0238) groups, than these two wild groups had with each other (*F*_ST_ = 0.0442), suggesting that the domesticated lines may have been a key vector in the North American invasion history. The AMOVA (Table 6) revealed that variation was strongly partitioned within individuals (72.45%), rather than among individuals within groups (24.42%) or among groups (3.14%); again not surprising for an obligate outcrossing species.

**Table 6:** Analysis of molecular variance and population pairwise *F*_ST_ values among three groups (Group 1 CC—chicory cultivars, Group 2 EU—wild Eurasian chicory, Group 3 NA—North American chicory from Arlequin v. 3.5.1.3

AMOVA design and results				
Source of variation	*df*	Sum of squares	Variance components	Percentage of variation
Among populations	2	51.662	0.10672	3.14
Among individuals within populations	589	2430.050	0.83060	24.42
Within individuals	592	1459.000	2.46453	72.45
Total	1183	3940.712	3.40184	
Fixation Indices: *F*_IS_ = 0.25207^a^, *F*_ST_ = 0.03137^a^, *F*_IT_ = 0.27553^a^				

Group pairwise *F*_ST_ values			
	1CC	2EU	3NA
1CC	0.00000		
2EU	0.02540^a^	0.00000	
3NA	0.02381^a^	0.04423^a^	0.00000

a Significant values at *p* < .05 after 1023 permutations.

**Figure 3:**
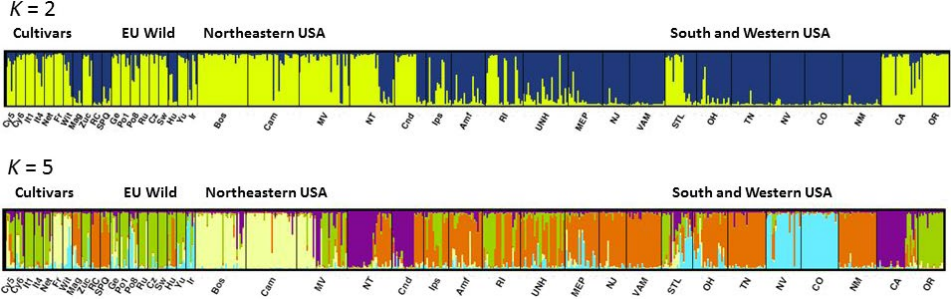
STRUCTURE analysis based on 12 SSR markers for the combined collection of 20 Eurasian and 20 North American chicory accessions, assuming *K* = 2 and 5

Four genetic groups for Eurasian chicory and cultivars were resolved by the microsatellite data analysis and individuals in several populations showed evidence of admixture (Figure 1). We conducted a partial STRUCTURE analysis for just the 120 sampled plants of the 11 CC and 9 EU populations, and the number of clusters (*K*) was varied from one‐seven. The highest likelihood in the partial analysis was obtained when *K* was set to four, and also the maximal Δ*K* occurred at *K* = 4 using the method of Evanno et al. (2005) (Appendix S3). In the STRUCTURE analysis for all 592 sampled plants, the number of clusters (*K*) was varied from one to twelve. Using the method of Evanno et al. (2005), the maximal Δ*K* occurred at *K* = 2, with the next largest peak at *K* = 5 (Figure 3, Appendix S4).

The chicory cultivars grouped into three of the five major clusters. Root and Catalogna chicory shared their genetic ancestry, Witloof chicory cultivars formed one group. We could detect gene flow between cultivars, and apparent mixed‐ancestry was clearly visible in a primitive cultivar Cy6 and in Radicchio varieties (It4, Net). Wild Eurasian populations showed mosaic genotypes, and no clear nuclear genotype division was observed between northern and southern populations as was detected for the chloroplast data. Radicchio (It1) and Pain de Sucre (Zuc) genetic cluster was present in Ge, Po1, Ru, Cz, Yu, and Ir populations (Figure 1).

North American populations include all five genetic groups and show admixture between them. Some populations seem to be very similar to specific domesticated lines. BOS and CAM match well to the Witloof accessions Fr and Wit, NJ and VAM are close to Catalogna accessions RC and SPQ and OR is similar to the Radicchio accessions It1, It4, and Net. Interestingly, two western populations, NV and CO, seem most similar to the wild population from Iran (Ir). The most widespread genetic group, Catalogna, is present throughout the east but highly represented in only NM in the west. One rather dominant genetic group in three of the NA populations (NT, Cnd, and CA) does not seem to be well represented in the CC or EU collections. Several North American populations showed multiple distinct genetic clusters within a population (MV, NT, Cnd, Ips, RI, and CA). Mosaic genotypes suggesting high levels of gene flow were detected in MV, Amf, UNH, MEP, STL, and OH populations. The presence of apparent mixed‐ancestry is clearly visible in urban areas with disturbed habitats. Populations with single ancestry seem more common in less disturbed more natural sites such as deserts and nature reserves (NJ, VAM, TN, NV, CO, NM; Figures 2 and 3).

## 4 Discussion

The present study is a first broad genetic survey of North American chicory placed into a global framework of the species’ natural history. The genetic diversity for the 12 SSR markers was high (*H*_e_ = 0.61), similar to another North American non‐native taxon in the Asteraceae (Eriksen et al., 2014) and higher then mean values (0.56) from a recent compilation of 1512 species of Asteriods (Merritt, Culley, Avanesyan, Stokes, & Brzyski, 2015). cpDNA variation in populations is generally low, but it serves as a useful tool for monitoring seed dispersal and maternal contributions (Ennos, Sinclair, Hu, & Langdon, 1999; Wallace et al., 2011). In chicory, cpDNA haplotype diversity was low as expected, and the different native and domesticated sources of seed generally possessed a single cpDNA haplotype. Significantly, we detected all three haplotypes from native European collections within North America. Together, chloroplast and nuclear data provided evidence of multiple introductions and admixture; three different cpDNA haplotypes suggest different seed sources and unique genotypes, high nuclear genetic diversity, and high intergroup gene flow suggest hybridization and recombination.

Domestication of wild plants is considered a long process that starts with human selection. Breeding and cultivation of these plants terminates in a fixation of favored morphological and genetic differences distinguishing a domesticate from its wild progenitor (Pickersgill, 2007). A subset of weeds and invasive plants has evolved in the reverse direction from domesticated ancestors by at least two different pathways. In California where no wild relative existed, weedy rye appears to have evolved directly from the escaped crop (Burger, Lee, & Ellstrand, 2006). A different pathway is represented by Europe's weed beet (Desplanque et al., 1999), which descended from hybrids between a crop and a wild ancestor. Chicory in the invaded region appears to be similar to the weedy rye story except that there was not a single origin or crop ancestor; we have observed different genetic clusters and different cpDNA haplotypes in North America that are similar to different chicory cultivars. In addition, the presence of new mosaic hybrid genotypes and a relatively high number of private alleles suggests hybridization among different escaped domesticated lines and weedy lines which may have dispersed naturally or as contaminants of seed stocks. Added to this is the nearly 250 years of history within the North America, creating a complex evolutionary picture.

The domesticated accessions in our study do not show reduced allelic diversity in accordance with previous studies (Kiaer et al., 2009; Van Cutsem et al., 2003). Independent domestication efforts leading to different chicory cultivar groups were suggested previously based on the genetic patterns of wild and cultivated chicory in Europe (Kiaer et al., 2009) and our results also confirm this both broadly and in within specific lineages. Broadly speaking, multiple cpDNA haplotypes from wild Eurasian populations were found in the current chicory cultivars indicating a variety of origins. Within specific and agronomically important cultivar groups such as Witloof, Radicchio, and Catalogna, different wild accessions often seem to have a close affinity to one or the other of these. The Witloof group was bred to be strongly uniform, likely resulting in a narrowing of its genetic base (de Proft, Van Stallen, & Veerle, 2003). Our data confirm this suggestion, as Witloof accessions had the lowest polymorphism and observed heterozygosity and highest inbreeding coefficient among cultivated accessions. Witloof and root chicories were shown to be in closely related clusters using AFLP data analysis (Kiers et al., 2000). It was suggested that Witloof chicory is derived from the Magdeburger root chicory type (Bellamy et al., 1996) and our microsatellite data showed Witloof to be an extraction of the more diverse Magdeburger root chicory, supporting this hypothesis. The Radicchio accessions are cultivated for their leaves and showed wide genetic variability. The literature lists a hybrid origin of the Radicchio type Chioggia as a result of crosses between chicory and an endive cultivar (Barcaccia et al., 2003), but this hypothesis could not be either confirmed or rejected by previous genetic studies (Kiers et al., 2000; McDade, 1997). The presence of endive cpDNA haplotype detected in Radicchio “Variegata Di Chioggia” (It1) accession supports the interspecific hybrid origin of Radicchio.

Chicory was introduced into North America as a food and fodder crop around the time of the American Revolution. Fodder accessions are derived from leaf chicories, which are well represented in our collection of domesticated lines; however, additional accessions not included in our collection may have contributed to the invasion, particularly in pastures and prairies in the Western USA. The absence in our analysis of some specific domesticated lines or weed contaminants in seed lots could explain some of the unique alleles and the unique genetic groups. However, few of the private alleles detected reached a high frequency in a given population; the highest frequency was 0.26 for one allele in one population (TN) and only three others exceeded 0.10. Thus, we have no indication that we missed a major contributor to the North American invasion.

While a high genetic diversity is not necessarily a prerequisite for successful habitat colonization (Ward, Gaskin, & Wilson, 2008), invaders with greater genetic diversity may be able to adapt more readily to new environments. Analysis of allelic diversity of microsatellite data revealed no reduction of genetic diversity in wild, introduced populations versus domesticated lines. Bottlenecks were not expected, given the outcrossing breeding system, and the dispersal history in North America spanning over two hundred years since its introduction. Recent genetic evidence implies that many large‐scale plant colorizations were associated with multiple introductions, and a bottleneck would be inferred only if introduced populations contain fewer rare alleles than expected (Peery et al., 2012). Furthermore, simulations indicate that even moderate gene flow can mitigate the detection of genetic bottlenecks using traditional methods (Fitzpatrick et al., 2012), and extensive admixture was evident in our STRUCTURE analysis.

Our results are consistent with previous studies in European chicory which showed high levels of gene flow between cultivated and wild chicory accessions (Kiaer et al., 2009; Van Cutsem et al., 2003). As a group, the domesticated accessions in this study were as similar to wild Eurasian accessions (*F*_ST_ = 0.0254) as they were to wild NA accessions (*F*_ST_ = 0.0238). Conversely, the wild accessions of Eurasia and wild NA accessions showed nearly twice the *F*_ST_ value (0.0442). This suggests that North American chicory evolved primarily from introduced domesticated lines over the past two hundred years. However, some NA populations (NV and CO) seemed to have a closer affinity to wild accessions from EU and other populations in NA seemed relatively distinct from the major domesticated lines surveyed in this study. This together with the high private allelic richness found in the invaded region indicate additional introductions and sources of diversity as well as the possibility of local adaptation to the new environments.

Our NA collection of populations did cover a broad spectrum of habitats with different histories. One of the sampled populations was collected along the roadsides leading to Tufton Farm, the location of the first recorded chicory planting in the United States by Thomas Jefferson. Others came from rural and likely more recent introductions such as those in Colorado, where it is labeled a noxious weed, Nevada and New Mexico. The structural analysis showing the similarity of some of these populations (CO, NV) to that in the Iran region of the EU collection as well as some of the highest private allelic richness scores (NV, NM) could indicate the potential for adaptation for drier habitats or other local conditions. Mosaic genotypes detected in urban populations may imply multiple introductions and an extensive gene flow in these areas.

There are no previous studies examining genetic diversity and population structure in North American chicory. The objective was to determine the genetic relationships and structuring between domesticated, wild, and introduced *Cichorium intybus* populations. Chicory possesses many traits that contribute to its ability to spread and adapt to a variety of habitats. These favorable traits include a self‐incompatibility system that promotes outcrossing, plasticity, tolerance of different environmental conditions, and also recently discovered allelopathy (Wang et al., 2012). Clearly, the cultivar genetic groups are major contributors to the populations, but some NA populations are unique, have multiple origins, and likely have evolved as they adapt to these new habitats. Chicory cultivars may have come to the United States well equipped for a successful invasion, thanks to their agriculturally desirable traits selected during their domestication. Kiaer, Philipp, Jørgensen, and Hauser (2007) compared fitness traits of wild and cultivated chicory plants and showed that chicory cultivars produced more seeds and flowered longer than wild chicory accessions. These biological features certainly enhanced chicory's ability to spread and persist in the new habitat.

## Conflict of Interest

None declared.
