# Supplementary material for: Population structure in chicory (Cichorium intybus): A successful U.S. weed since the American revolutionary war
Source: Ecol Evol. 2017 May 2;7(12):4209–19. doi: 10.1002/ece3.2994 (PMC5478081; doi:10.1002/ece3.2994)
Supplement: Supplementary file 1 [file ECE3-7-4209-s001.docx]

APPENDICES

Appendix S1. Allelic Richness (HP-Rare) for *C. intybus* populations

AVG OVER LOCI 5291 3984 5055 12770 6865 1385 11019 13676 2050 3899 7179 8271

Cy5 2.75 4.0000 3.0000 2.0000 4.0000 1.0000 4.0000 1.0000 3.0000 4.0000 3.0000 2.0000 2.0000

Cy6 3.67 2.0000 3.0000 3.0000 5.0000 5.0000 3.0000 2.0000 4.0000 5.0000 5.0000 3.0000 4.0000

It1 4.67 7.0000 5.0000 3.0000 5.0000 5.0000 4.0000 4.0000 4.0000 4.0000 5.0000 5.0000 5.0000

It4 3.58 4.0000 4.0000 4.0000 3.0000 4.0000 3.0000 3.0000 4.0000 4.0000 3.0000 4.0000 3.0000

Net 3.92 6.0000 4.0000 4.0000 5.0000 6.0000 3.0000 3.0000 1.0000 3.0000 5.0000 5.0000 2.0000

Fr 2.17 3.0000 3.0000 2.0000 2.0000 1.0000 3.0000 2.0000 2.0000 1.0000 2.0000 2.0000 3.0000

WT 2.00 5.0000 3.0000 1.0000 1.0000 1.0000 2.0000 1.0000 1.0000 3.0000 2.0000 1.0000 3.0000

MAG 3.17 7.0000 2.0000 2.0000 3.0000 3.0000 4.0000 4.0000 3.0000 4.0000 2.0000 2.0000 2.0000

Ge4Z 3.67 4.0000 5.0000 4.0000 5.0000 4.0000 2.0000 3.0000 3.0000 5.0000 3.0000 3.0000 3.0000

RC 3.50 8.0000 2.0000 4.0000 3.0000 5.0000 4.0000 2.0000 5.0000 3.0000 2.0000 1.0000 3.0000

SPQ 3.33 9.0000 4.0000 1.0000 4.0000 4.0000 2.0000 1.0000 4.0000 2.0000 3.0000 4.0000 2.0000

Ge1 4.33 5.0000 5.0000 4.0000 6.0000 6.0000 3.0000 3.0000 3.0000 5.0000 3.0000 5.0000 4.0000

Po1 6.75 8.0000 5.0000 3.0000 0.0000 5.0000 3.0000 5.0000 5.0000 3.0000 6.0000 5.0000 3.0000

Po8 2.92 4.0000 2.0000 4.0000 4.0000 3.0000 2.0000 3.0000 1.0000 5.0000 3.0000 2.0000 2.0000

Ru 3.92 6.0000 5.0000 4.0000 4.0000 6.0000 3.0000 3.0000 3.0000 4.0000 4.0000 3.0000 2.0000

Sw 2.25 5.0000 3.0000 2.0000 2.0000 3.0000 2.0000 1.0000 2.0000 2.0000 1.0000 2.0000 2.0000

Hu 3.42 6.0000 2.0000 5.0000 4.0000 5.0000 3.0000 1.0000 2.0000 4.0000 4.0000 2.0000 3.0000

Yu 2.92 4.0000 4.0000 3.0000 2.0000 2.0000 2.0000 4.0000 3.0000 3.0000 3.0000 3.0000 2.0000

Ir 4.17 8.0000 5.0000 2.0000 5.0000 4.0000 3.0000 4.0000 2.0000 6.0000 4.0000 4.0000 3.0000

Cz 4.17 8.0000 4.0000 5.0000 4.0000 5.0000 2.0000 4.0000 2.0000 4.0000 5.0000 3.0000 4.0000

Bos 3.60 5.7466 2.5527 3.5844 4.0590 4.8294 4.7974 1.9966 1.9976 3.7976 4.2034 2.8646 2.8292

Cam 3.52 4.8671 2.1858 4.8565 4.2689 4.3143 5.5220 1.3750 1.5739 3.7989 2.7759 2.8294 3.8644

MV 3.81 3.7451 3.7597 4.1361 3.7955 4.0519 4.8930 2.1505 2.8949 3.6872 3.7542 3.6321 5.1669

NT 3.52 3.8094 3.3999 3.3904 4.8704 4.7255 5.1045 2.1628 1.0000 3.9879 3.2887 3.0216 3.4961

Cnd 3.35 4.8029 1.8506 2.5376 5.5402 2.9593 3.8512 3.1864 1.0000 4.0430 3.5414 2.8352 4.0541

Ips 3.53 5.3072 2.8650 4.0000 4.5238 2.0000 4.7374 3.7994 2.7642 3.5922 4.0761 2.2980 2.3748

Amf 4.04 7.2459 3.1176 5.4750 4.3685 4.3686 4.7793 2.9362 3.8352 3.0534 3.5313 3.2907 2.4943

RI 4.12 4.7109 3.6728 4.7941 4.0062 5.2525 4.5968 3.8625 3.2569 4.1481 4.6229 2.8945 3.5666

UND 4.27 6.6504 4.2545 3.5526 4.6499 4.6003 5.1540 4.0723 3.9983 3.1189 4.4944 3.1652 3.4841

MEP 3.77 6.8796 2.2660 4.4128 4.2103 4.0779 4.8839 3.8981 2.6089 3.2501 3.6674 2.8224 2.2726

NJ 3.67 4.6891 2.4853 5.4975 4.0754 3.5432 4.0318 2.5800 6.1857 2.6480 3.8647 2.4118 2.0000

VAM 3.82 6.9263 2.9589 5.2746 3.9512 3.9193 4.6895 2.3844 3.5610 2.6148 3.6082 3.4723 2.4755

STL 4.52 6.6280 4.0830 3.8119 4.0654 5.5565 6.5411 3.5724 3.3537 4.0269 4.6084 3.4434 4.5498

OH 3.91 6.5341 2.9299 4.3745 6.0761 4.6075 5.4674 2.3669 1.8982 3.3714 3.3634 2.9683 2.9329

TN 3.12 5.8420 2.8911 3.3092 3.3946 4.1068 2.6526 1.0000 4.4699 2.2234 1.9999 2.0298 3.4669

NV 3.73 6.6384 2.8722 3.3507 4.8509 3.9646 6.1314 3.5288 1.6918 3.3672 3.4686 1.9999 2.8722

CO 3.03 7.1530 2.8600 2.9896 4.8679 3.0497 2.7238 2.2339 1.2500 3.2283 2.4999 2.2496 1.2500

NM 3.37 6.0802 2.6113 3.9096 4.2422 3.7783 4.0225 2.1768 3.7059 3.5369 1.6973 1.8372 2.8367

CA 3.43 3.3051 2.9229 2.7777 4.6705 4.6605 5.5481 1.4615 2.8905 4.0190 2.8231 2.6920 3.3851

OR 5.71 2.3721 3.1248 0.0000 6.8308 3.6610 1.6169 2.3871 3.5399 4.2150 3.2172 3.6060 3.9420

Appendix S2. Private allelic richness (HP-Rare) for *C. intybus* populations

AVG OVER LOCI 5291 3984 5055 12770 6865 1385 11019 13676 2050 3899 7179 8271

Cy5 0.00 0.0000 0.0000 0.0000 0.0000 0.0000 0.0000 0.0000 0.0000 0.0000 0.0000 0.0000 0.0000

Cy6 0.17 0.0000 0.0000 0.0000 0.0000 0.0000 0.0000 0.0000 0.0000 1.0000 1.0000 0.0000 0.0000

It1 0.12 0.0000 0.3808 0.0000 0.0000 0.0000 0.0000 0.0000 0.0000 0.0000 0.0000 0.0000 1.0000

It4 0.00 0.0000 0.0000 0.0000 0.0000 0.0000 0.0000 0.0000 0.0000 0.0000 0.0000 0.0000 0.0000

Net 0.00 0.0000 0.0000 0.0000 0.0000 0.0000 0.0000 0.0000 0.0000 0.0000 0.0000 0.0000 0.0000

Fr 0.00 0.0000 0.0000 0.0000 0.0000 0.0000 0.0000 0.0000 0.0000 0.0000 0.0000 0.0000 0.0000

WT 0.00 0.0000 0.0000 0.0000 0.0000 0.0000 0.0000 0.0000 0.0000 0.0000 0.0000 0.0000 0.0000

MAG 0.01 0.0000 0.0000 0.0000 0.0000 0.0000 0.1571 0.0000 0.0000 0.0000 0.0000 0.0056 0.0000

Ge4Z 0.00 0.0000 0.0000 0.0000 0.0000 0.0000 0.0000 0.0000 0.0000 0.0000 0.0000 0.0000 0.0000

RC 0.11 0.0000 0.0000 0.0000 0.0000 1.0000 0.0138 0.3175 0.0161 0.0000 0.0000 0.0000 0.0000

SPQ 0.06 0.0000 0.0000 0.0000 0.0000 0.0000 0.0000 0.0000 0.0000 0.0000 0.0000 0.7143 0.0000

Ge1 0.03 0.0000 0.0000 0.0000 0.0000 0.3409 0.0000 0.0000 0.0000 0.0000 0.0000 0.0000 0.0000

Po1 0.01 0.0000 0.0000 0.0000 0.0000 0.0000 0.0000 0.0000 0.1219 0.0000 0.0000 0.0000 0.0000

Po8 0.00 0.0000 0.0000 0.0000 0.0000 0.0000 0.0000 0.0000 0.0000 0.0000 0.0000 0.0000 0.0000

Ru 0.00 0.0000 0.0000 0.0000 0.0000 0.0000 0.0000 0.0000 0.0000 0.0000 0.0000 0.0000 0.0000

Sw 0.00 0.0078 0.0000 0.0000 0.0000 0.0000 0.0000 0.0000 0.0000 0.0000 0.0000 0.0000 0.0000

Hu 0.02 0.0000 0.0000 0.0000 0.0000 0.0000 0.1813 0.0000 0.0000 0.0000 0.0000 0.0000 0.0000

Yu 0.00 0.0000 0.0000 0.0000 0.0000 0.0000 0.0000 0.0000 0.0000 0.0000 0.0000 0.0000 0.0000

Ir 0.00 0.0000 0.0000 0.0000 0.0000 0.0000 0.0000 0.0000 0.0000 0.0000 0.0000 0.0000 0.0000

Cz 0.04 0.0238 0.0000 0.0000 0.0000 0.0000 0.0000 0.4778 0.0000 0.0000 0.0000 0.0000 0.0000

Bos 0.04 0.0000 0.0000 0.0000 0.0000 0.1875 0.3144 0.0000 0.0000 0.0000 0.0000 0.0000 0.0000

Cam 0.02 0.0000 0.0000 0.0000 0.0000 0.0000 0.2667 0.0000 0.0000 0.0000 0.0000 0.0000 0.0000

MV 0.05 0.1525 0.0000 0.0000 0.0000 0.0000 0.0000 0.0000 0.0000 0.0000 0.0000 0.1653 0.2396

NT 0.01 0.0000 0.0000 0.0000 0.0000 0.0000 0.0002 0.1339 0.0000 0.0000 0.0000 0.0009 0.0000

CND 0.04 0.0000 0.0000 0.0000 0.0000 0.0000 0.0004 0.0000 0.0000 0.0000 0.0000 0.0000 0.5154

IP 0.00 0.0000 0.0000 0.0000 0.0000 0.0000 0.0000 0.0000 0.0000 0.0000 0.0000 0.0000 0.0000

AMF 0.09 0.0000 0.0000 0.0000 0.0000 0.0000 0.0995 0.0000 0.9890 0.0000 0.0000 0.0000 0.0000

RI 0.05 0.1161 0.0000 0.0000 0.0000 0.0000 0.0001 0.0000 0.0000 0.0000 0.4415 0.0010 0.0000

UND 0.02 0.0950 0.0000 0.0000 0.0000 0.0000 0.0941 0.0000 0.0000 0.0000 0.0000 0.0303 0.0000

MEP 0.01 0.1172 0.0000 0.0000 0.0000 0.0000 0.0002 0.0000 0.0000 0.0000 0.0000 0.0000 0.0000

NJ 0.07 0.0000 0.0000 0.0000 0.0000 0.0000 0.0002 0.0000 0.8473 0.0000 0.0000 0.0000 0.0000

VAM 0.06 0.1172 0.0000 0.0000 0.0000 0.0000 0.6278 0.0000 0.0008 0.0000 0.0000 0.0000 0.0000

STL 0.07 0.0000 0.0000 0.0000 0.0000 0.0000 0.3740 0.0000 0.0000 0.0000 0.0000 0.0000 0.4973

OH 0.10 0.0000 0.0000 0.0000 0.0000 0.0000 0.5458 0.6696 0.0000 0.0000 0.0000 0.0067 0.0000

TN 0.14 0.0000 0.0000 0.0000 0.0000 0.0000 0.0000 0.0000 1.6724 0.0000 0.0000 0.0111 0.0000

NV 0.33 2.4149 0.0000 0.0000 0.0000 0.0000 0.3290 1.1765 0.0000 0.0000 0.0000 0.0000 0.0000

CO 0.06 0.6973 0.0000 0.0000 0.0000 0.0000 0.0473 0.0000 0.0000 0.0000 0.0000 0.0000 0.0000

NM 0.24 1.5198 0.0000 0.0000 0.0000 0.2500 0.2501 0.0000 0.9020 0.0000 0.0000 0.0000 0.0002

CA 0.04 0.0000 0.0000 0.0000 0.0000 0.0000 0.4261 0.0000 0.0000 0.0000 0.0000 0.0949 0.0000

OR 0.10 0.0000 0.0000 0.0000 0.0000 0.0000 0.0000 0.0000 0.0000 0.0000 0.3750 0.8067 0.0000
