# Supplementary figures and images for: Population structure in chicory (Cichorium intybus): A successful U.S. weed since the American revolutionary war
Source: Ecol Evol. 2017 May 2;7(12):4209–19. doi: 10.1002/ece3.2994 (PMC5478081; doi:10.1002/ece3.2994)

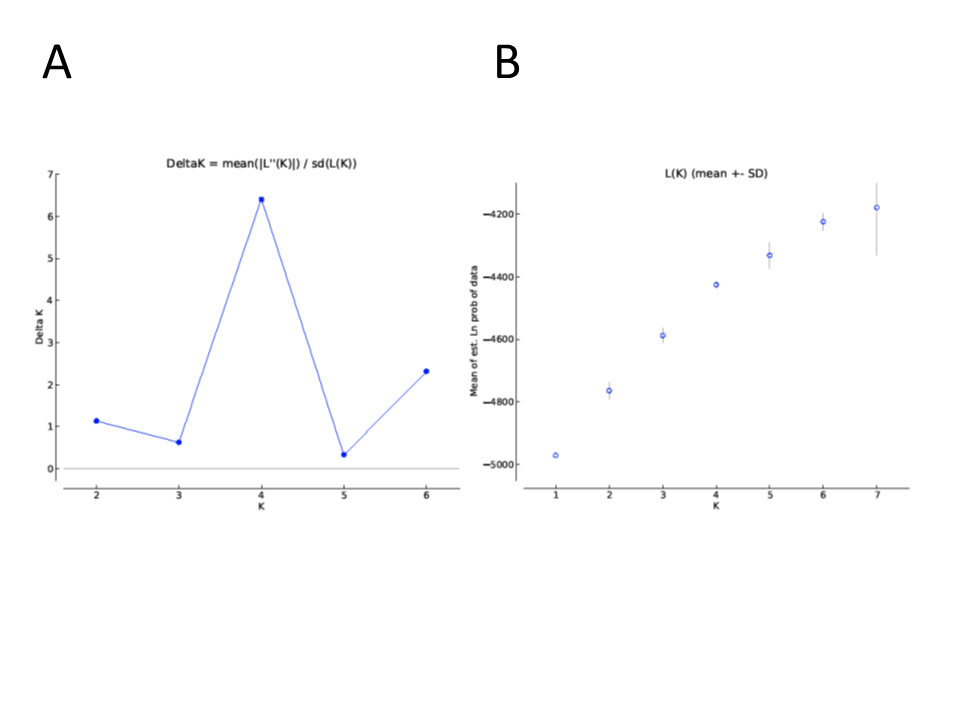

Supplement: Supplementary file 2 [file ECE3-7-4209-s002.tif]

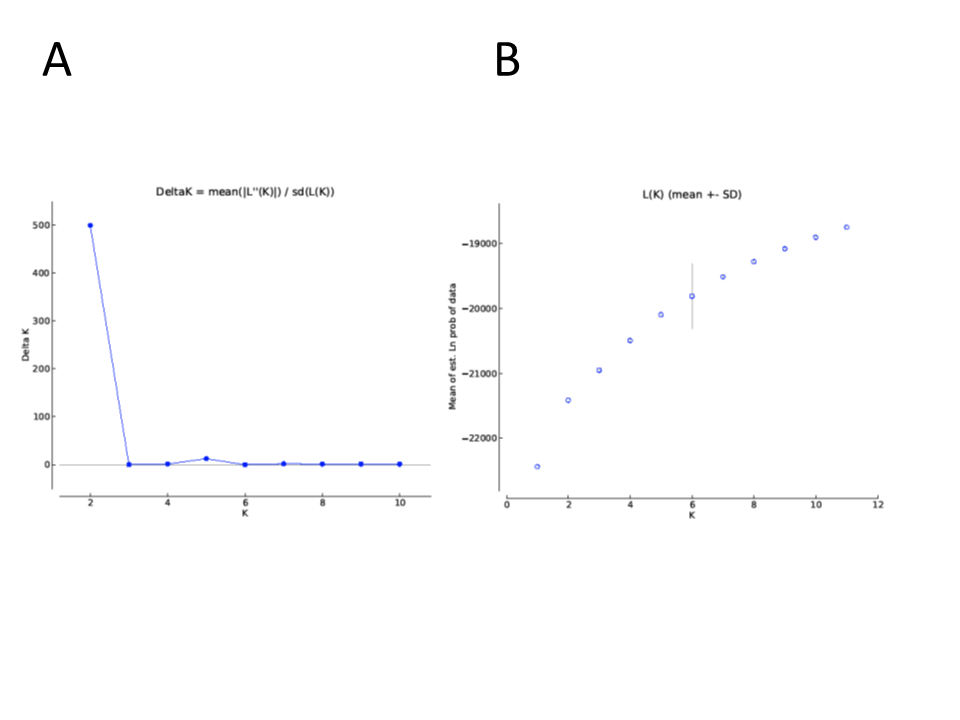

Supplement: Supplementary file 3 [file ECE3-7-4209-s003.tif]
